# Supplementary material for: Improving Small Molecule pK a Prediction Using Transfer Learning With Graph Neural Networks
Source: Front Chem. 2022 May 26;10:866585. doi: 10.3389/fchem.2022.866585 (PMC9204323; doi:10.3389/fchem.2022.866585)
Supplement: Supplementary file 1 [file DataSheet1.pdf]

Improving Small Molecule pK_a Prediction Using Transfer Learning with Graph Neural Networks

Fritz Mayr (0000-0002-6621-2108)[1+], Marcus Wieder* (0000-0003-2631-8415)[1+], Oliver Wieder (0000-0003-4967-7613)[1], Thierry Langer (0000-0002-5242-1240)[1]

[+]contributed equally to this work; [1]Department of Pharmaceutical Sciences, Pharmaceutical Chemistry Division, University of Vienna, Althanstrasse 14, 1090 Vienna, Austria

***For correspondence:**
marcus.wieder@univie.ac.at (MW)

Supplementary Information

**Atom features**	
atomic number:	1, 6, 7, 8, 9, 15, 16, 17, 33, 35, 53
formal charge	-2,-1, 0, 1,2
hybridization:	1, 2, 3, 4
total number of Hs:	0, 1, 2, 3
explicit number of Hs:	0, 1, 2, 3
aromatic tag:	True, False
total valence:	1, 2, 3, 4, 5, 6
total degree:	1, 2, 3, 4
ring:	True, False
amide center atom:	True, False
SMARTS pattern:	one-hot-encoding of common acid-base pair substructures obtained from https://github.com/mcs07/MolVS

Table S.I.1. List of one-hot-encoding of atom features used for the node feature vector deposited in the node feature matrix X.

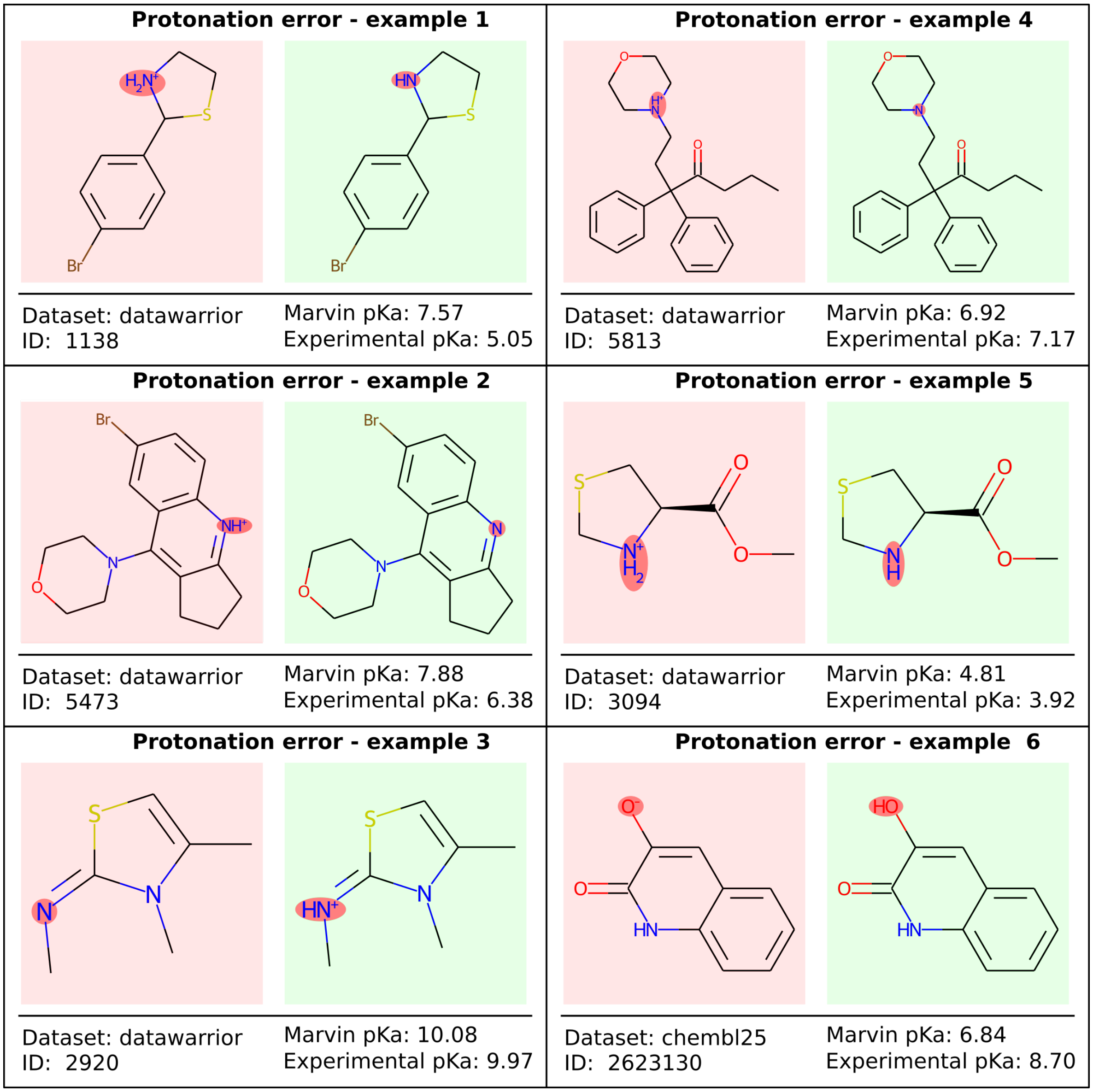

Figure S.I.1. Protonation state errors in the experimental data set. This exemplary selection shows molecules from the experimental data set provided by **?**] for which the protonation state provided does not correspond to the state at pH 7.4. For examples 1, 2, 4 and 5 with experimental pK_a values below 7.4 protonation at the reaction center would result in highly unlikely pentavalent nitrogen. For example 3 and 6 with pK_a values above 7.4 deprotonation at the reaction site can not be performed because of the lack of a suitable hydrogen. These error were corrected during our data preparation.

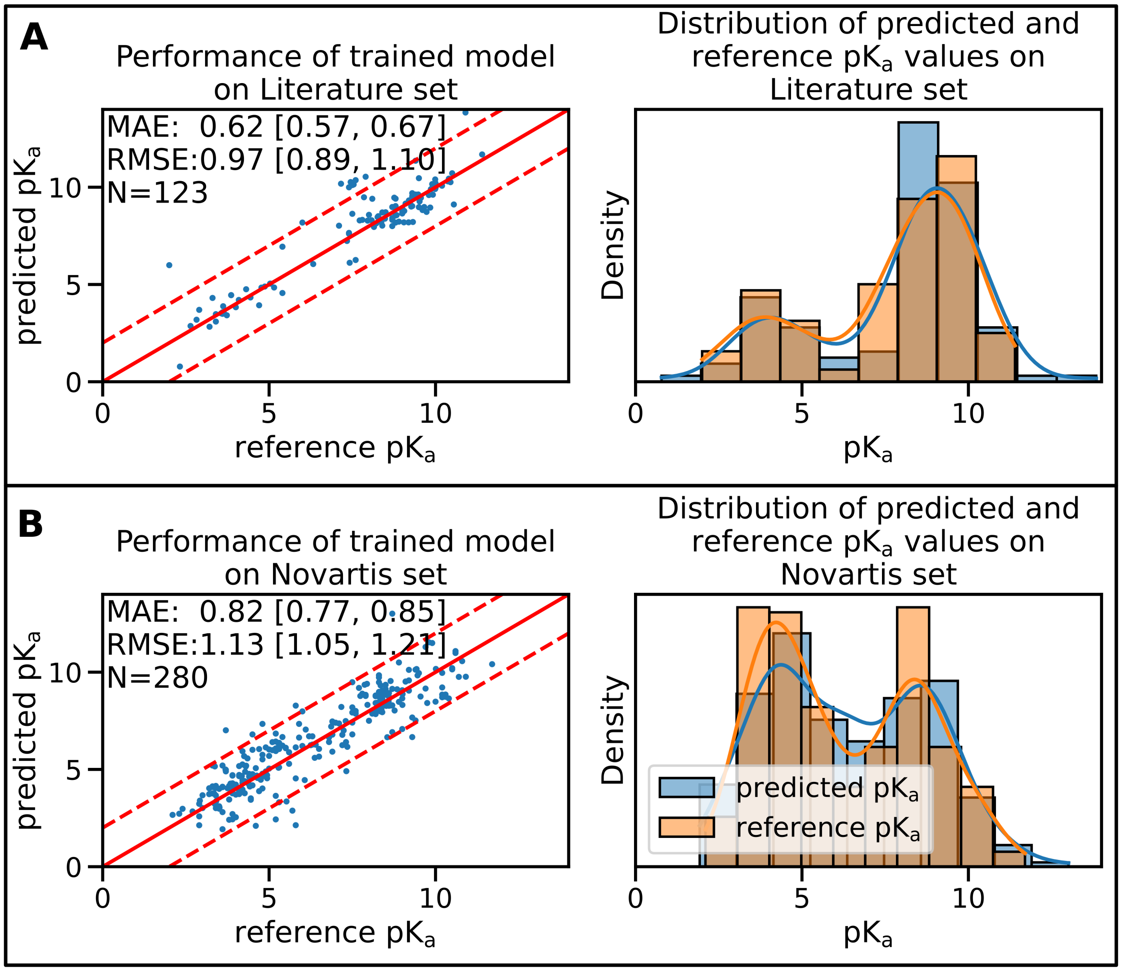

Figure S.I.2. Performance of the pre-trained GNN model on the Novartis and Literature test set is shown. 50 training runs with different training/validation splits were performed and for each training run the best model was selected based on its performance on the validation set (shown here is a single, randomly selected training run). Panel **A** shows the performance of the GNN model on the Literature data set. Panel **B** shows the performance of the GNN model on the Novartis data set. The solid red line in the scatter plot indicates the ideal behavior of the reference and predicted pK_a values, the dashed lines mark the ± 1 pk_a unit interval. Mean absolute error (MAE) and root mean squared error (RMSE) are shown, the values in bracket indicate the 90% confidence interval calculated from 50 repetitions with random training/validation splits. N indicates the number of investigated samples.

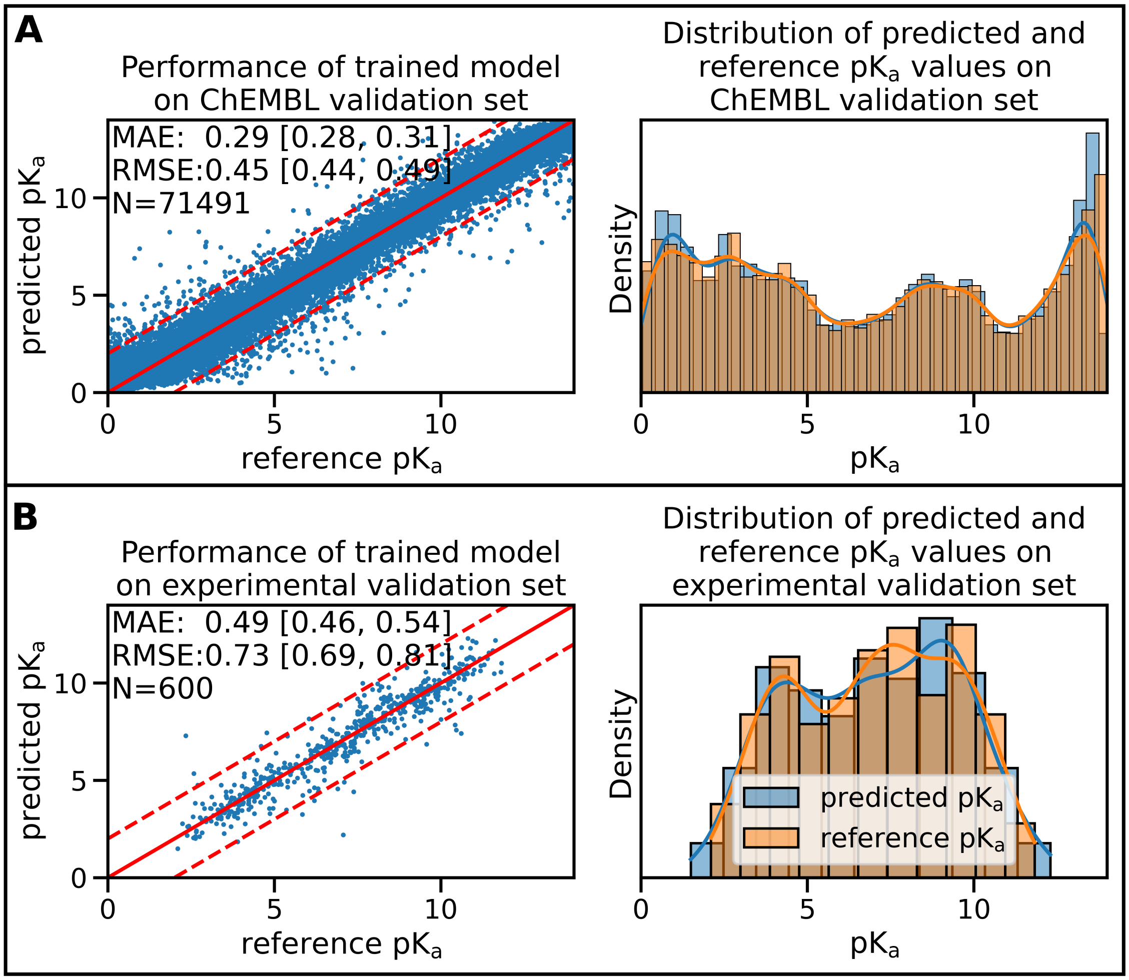

Figure S.I.3. Performance of the pre-trained and fine-tuned models are shown on the respective validation sets. 50 training runs with different training/validation splits were performed and for each training run the best model was selected based on its performance on the validation set (shown here is a single, randomly selected training run). Panel **A** shows the validation set performance of the best GNN model trained on the ChEMBL data set. Panel **B** shows the validation set performance starting from the same pre-trained model after fine-tuning on the experimental training set. The solid red line in the scatter plot indicates the ideal behavior of the reference and predicted pK_a values, the dashed lines mark the ± 1 pk_a unit interval. Mean absolute error (MAE) and root mean squared error (RMSE) are shown, the values in bracket indicate the 90% confidence interval calculated from 50 repetitions with random training/validation splits. N indicates the number of investigated samples.

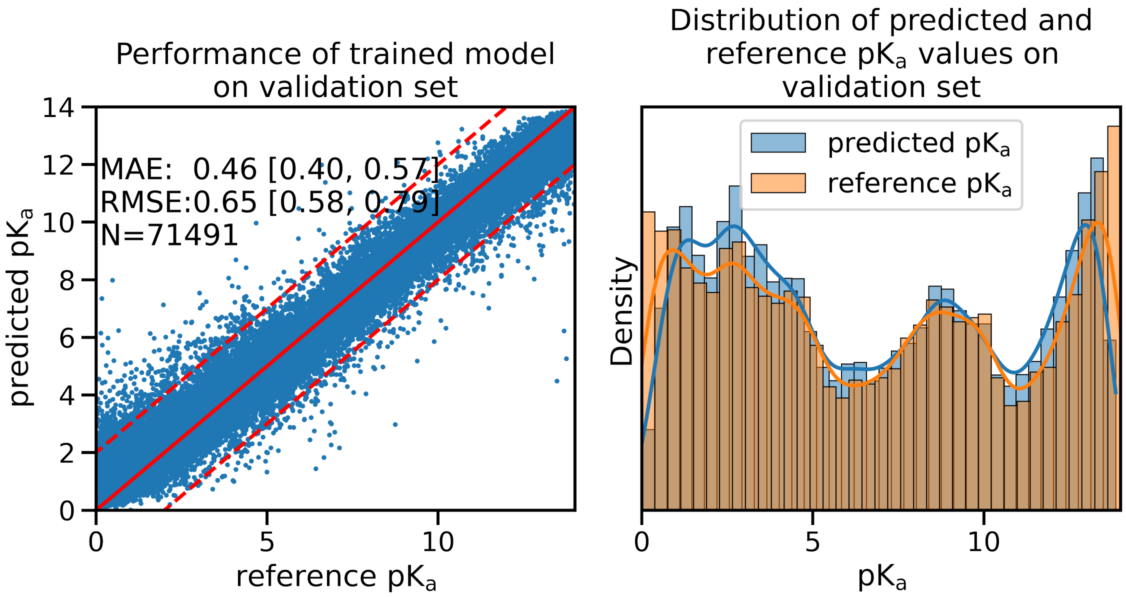

Figure S.I.4. The accuracy of the fine-tuned GNN model only decreases slightly when molecules from the ChEMBL data set are used for regularization. 50 fine-tuning runs with different training/validation splits were performed, each initialized using the parameters of 50 pre-training runs, and for each training run the best model was selected based on its performance on the validation set. In order to generate a single plot we selected randomly a single fine-tuning run and generated the scatter plot with the best performing model on the validation set. The solid red line in the scatter plot indicates the ideal behavior of the reference and predicted pK_a values, the dashed lines mark the ± 1 pk_a unit interval. Mean absolute error (MAE) and root mean squared error (RMSE) are shown, the values in bracket indicate the 90% confidence interval calculated from 50 repetitions with random training/validation splits. N indicates the number of investigated samples.

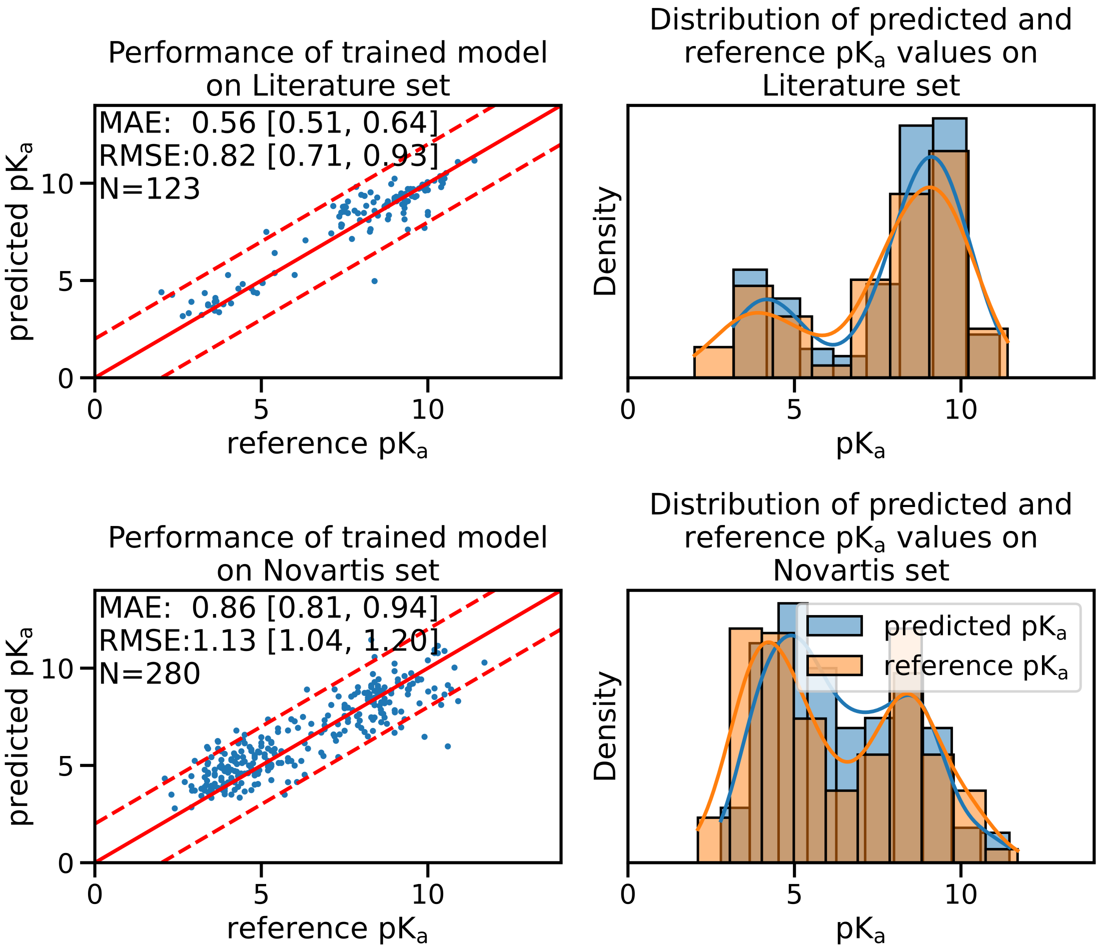

Figure S.I.5. The performance of the GNN model trained exclusively on the experimental data set is shown. 50 training runs with different training/validation splits were performed. To generate a single plot a randomly selected training run is shown. The solid red line in the scatter plot indicates the ideal behavior of the reference and predicted pK_a values, the dashed lines mark the ±1 pk_a unit interval. Mean absolute error (MAE) and root mean squared error (RMSE) are shown, the values in bracket indicate the 90% confidence interval calculated from 50 repetitions with random training/validation splits. N indicates the number of investigated samples.

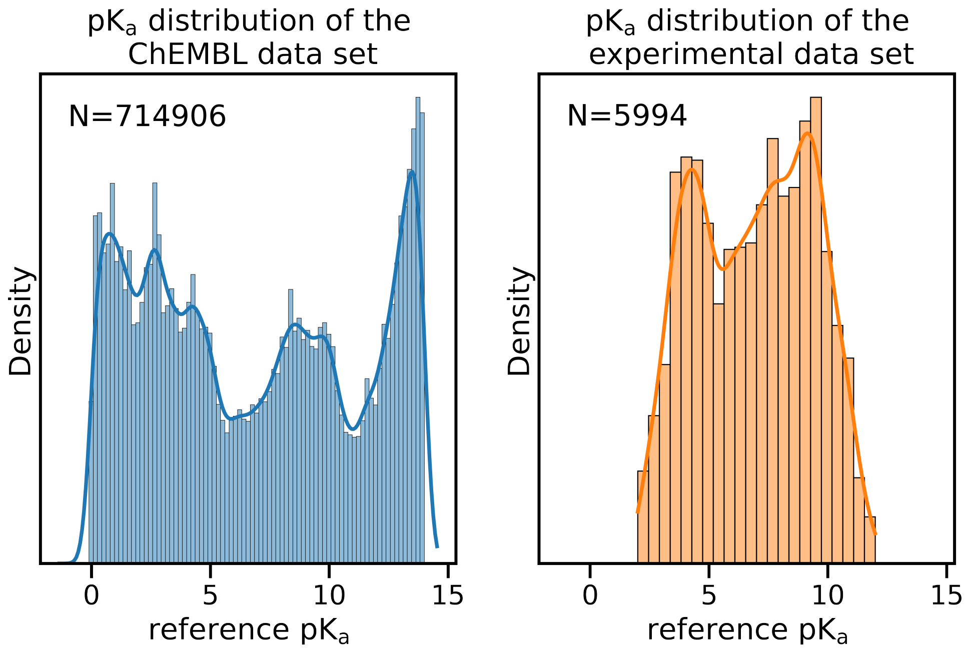

Figure S.I.6. The pk$_a$ distribution of ChEMBL and experimental data set are shown.

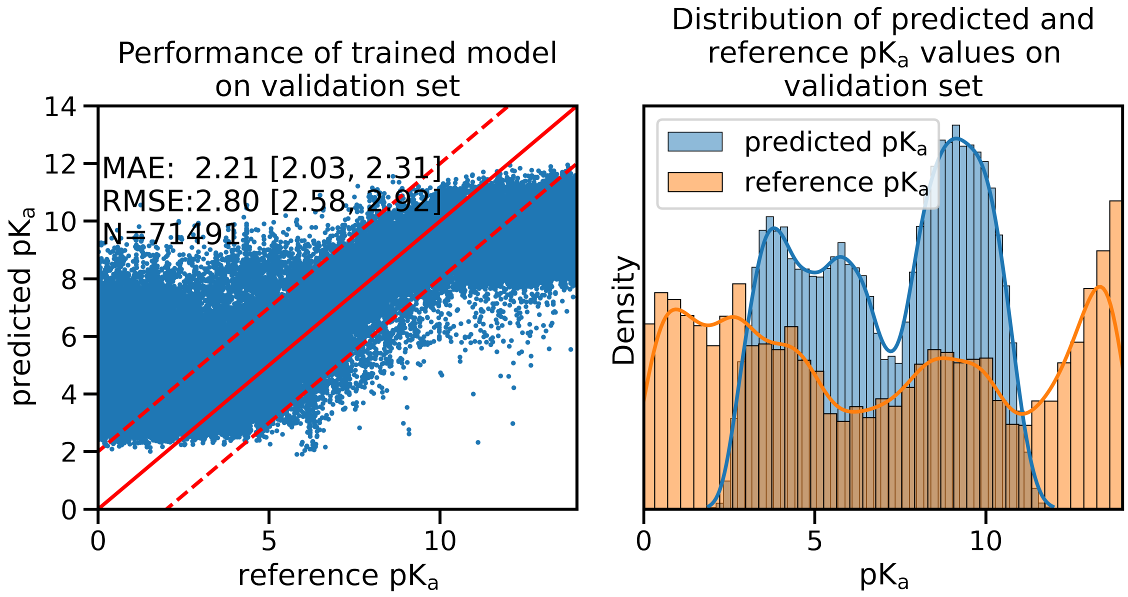

Figure S.I.7. The performance of the fine-tuned GNN model on the ChEMBL data set is shown. In contrast to the results obtained with the fine-tuned models shown in Figure S.I.4 the models shown here did **not** use regularization. The performance of the GNN model decreased significantly on the ChEMBL data, shifting pK$_a$ values above 12 and below 2. The solid red line in the scatter plot indicates the ideal behavior of the reference and predicted pK$_a$ values, the dashed lines mark the ± 1 pk$_a$ unit interval. Mean absolute error (MAE) and root mean squared error (RMSE) are shown, the values in bracket indicate the 90% confidence interval calculated from 50 repetitions with random training/validation splits. N indicates the number of investigated samples.

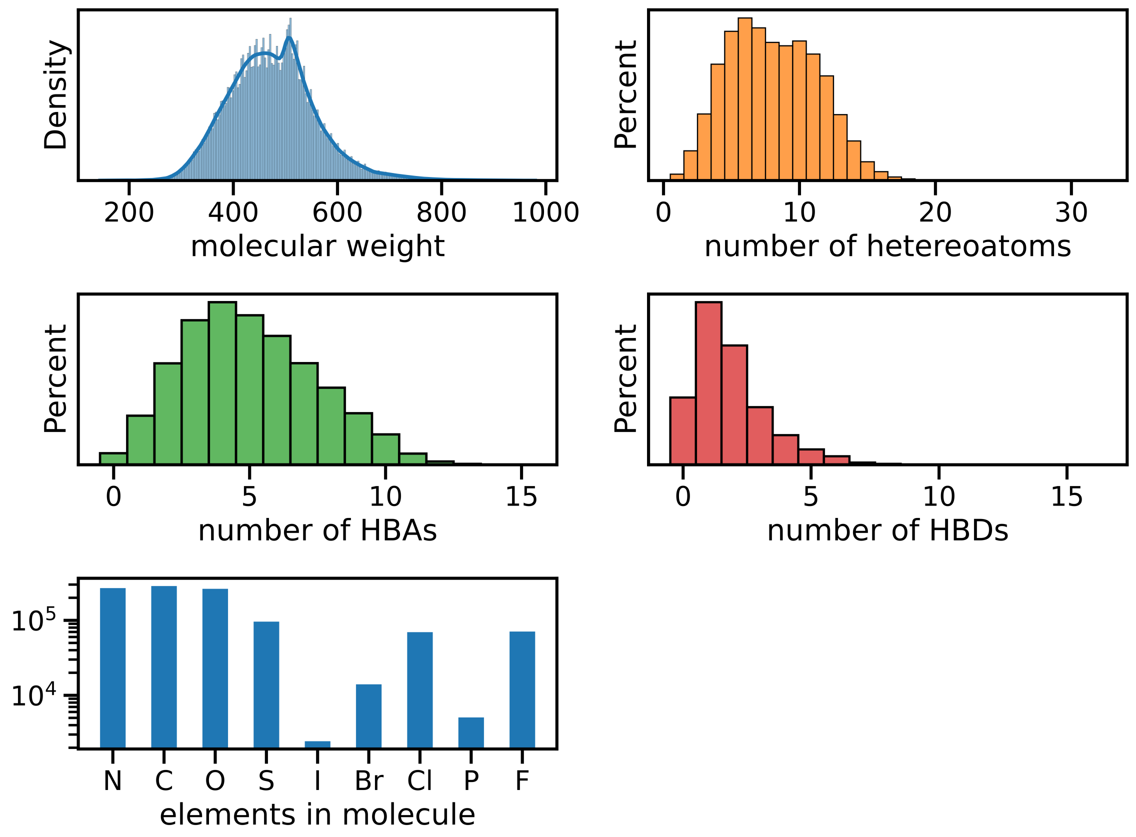

Figure S.I.8. The distribution of molecular weight, the number of hetereoatoms, hydrogen bond acceptors (HBAs) and hydrogen bond donors (HBDs) and distribution of elements per molecule are shown for the ChEMBL data set.

Predicting pK_a values

The protonation states and pK_a values discussed below are shown separately in the corresponding figures for each molecule. pK_a values are calculated as the average value of 50 trained models with the standard deviation in parenthesis. Values are rounded to one significant digit. Predictions are shown for `pkasolver-epic` (using transfer learning and the large ChEMBL data set for which pK_a values were calculated using `Epik`) and `pkasolver-light` (using *only* the fine-tuning data set for training). `pkasolver-epic` performs with higher accuracy than `pkasolver-light` for molecules with multiple protonation states. `pkasolver-light` performs well for monoprotic molecules, but its use is not recommended for molecules with multiple protonation states and/or sites.

Ethylenediaminetetraacetic acid

6 protonation state were identified and the calculated pK_a values are 1.5 (0.3), 1.9 (0.2), 2.3 (0.2), 2.6 (0.2), 6.1 (0.8), and 9.4 (0.4), shown in Figure S.I.9. Comparing this with the experimentally determined pK_a values of 0.0, 1.5, 2, 2.66, 6.16, and 10.24 the values are in good relative agreement and the correct protonation states are identified [**?**]. Only the first and last pK_a estimates are noticeable too high and too low respectively. This highlights a limitation of `pkasolver-epic`. The range of pK_a values present in the training data was between zero and 14, and predictions of pK_a values will stay in this range.

`pkasolver-light` identified the same first 5 protonation states (but misses the last one) and calculates the pK_a values with 2.8 (0.3), 3.1 (0.3), 3.5 (0.5), 5.1 (1.0) and 8.9 (0.7). While the states are correct the pK_a values of the first 2 states are too high.

Lisdexamfetamine

4 potential states were identified and the pK_a values calculated with 2.5 (1.4), 7.8 (0.2), 10.2 (0.1), and 13.2 (0.5), shown in Figure S.I.10. Estimated pKa values deposited in PubChem are 8.43, 10.21, and 15.89 [**?**]. `MolGpKa` identifies three protonation sites with pK_a values 8.3, 10.3, 13.8 (all of which agree qualitatively and quantitatively very well with the predicted values for `pkasolver-epic`). `pkasolver-epic` identified one additional protonation state, the charged state of the secondary amine with a pK_a value of 2.46 and a very high standard deviation of 1.4 pK_a units. While the protonation state is certainly possible at low pH, the high standard deviation points to uncertainty in the trained models.

`pkasolver-light` identified the same states and calculates the pK_a values with 7.10 (1.16), 8.38 (0.47), 9.15 (0.32) and 9.67 (0.42). The first state is significantly different than the value calculated with `pkasolver-epic`. Also the pK_a value of the last state is significantly too low.

Cocaine

A single protonation state was identified and `pkasolver-epic` calculates the pK_a values with 8.4 (0.1), shown in Figure S.I.11. This is in good agreement with the experimental pK_a value of 8.6 for the same protonation site [**?**].

`pkasolver-light` identified the same state and calculates the pK_a value with 8.5 (0.2).

Tyrosine

3 protonation states were generated and `pkasolver-epic` calculates the pK_a values with 2.4 (0.1), 9.1 (0.3), and 10.21 (0.2), shown in Figure S.I.12. The experimental pK_a values are 2.2, 9.21, and 10.5 [1]. This is one of the examples in which the sequential pK_a prediction seems to perform much better than the pK_a prediction for discrete groups, as performed e.g. by MolGpka (which calculates the pK_a values for the same protonation states with 2.3, 6.5, and 8.8).

`pkasolver-light` identified the carboxylic and amine group as possible protonation states and calculates the pK_a values with 4.85 (0.59) and 8.96 (0.63).

Taurine

A single protonation state was identified and `pkasolver-epic` calculates the pK_a values with 9.3 (0.4) (shown in Figure S.I.13), which agrees well with the experimental value of 9.3 [**?**].

[1] https://www.vanderbilt.edu/AnS/Chemistry/Rizzo/stuff/AA/AminoAcids.html

pkasolver-light identified the same state and calculates the pK_a value with 9.0 (0.6).

Aspergillic acid

2 protonation states were identified and pkasolver-epic calculates the pK_a values with 2.8 (0.5) and 4.6 (0.9), shown in Figure S.I.14. The experimental pK_a value is 5.5 [**?**]. Here, pkasolver provides the correct protonation states but for the protonation site with experimental pK_a value, the estimate is off by 1 pK_a unit.

pkasolver-light identified the same protonation states and calculates the pK_a values with 4.9 (0.6) and 5.9 (1.0). The experimentally determined value for the acid group is predicted accurately by pkasolver-light.

Ketamine

A single protonation site is identified and pkasolver-epic calculates the pK_a values with 7.3 (0.3), shown in Figure S.I.15. The experimental pKa value is 7.5 [**?**].

pkasolver-light identified the same state and calculates the pK_a value with 7.4 (0.4).

Levodopa

4 protonation site are identified and pkasolver-epic calculates the pK_a values with 2.4 (0.1), 9.0 (0.3), 9.9 (0.2) and 11.8 (0.5), shown in Figure S.I.16. Comparing this to the experimental pK_a values of 2.3, 8.7, 9.7, 13.42 shows good agreement [2]. This example shows again that when approaching extreme values (0 or 14) the model tends to perform worse than for values in the physiologically relevant range.

pkasolver-light identifies only the carboxylic acid and amine group as possible protonation sites and calculates the pK_a values with 5.6 (0.7) and 9.2 (0.5). The estimate for the carboxylic group is too low while the prediction for the amine is reasonably accurate.

Furosemide

5 protonation states are identified and pkasolver-epic calculates the pK_a values with 1.1 (0.4), 3.4 (0.3), 6.2 (1.1), 9.7 (0.1) and 13.1 (0.4), shown in Figure S.I.17. The pK_a value for the carboxylic group closely matches the experimentally measured pK_a of 3.52, also the pK_a value of the sulfonamide is in proximity to the experimental value of 7.5 [**?**]. There are no experimental values available for the more extreme pKa values but compared with e.g. MolGPka, the pK_a values for the protonation states are in good agreement.

pkasolver-light identifies the same protonation sites but different protonation states, which are shown in Figure S.I.18.

Arylguanidines

2 protonation states are identified and pkasolver-epic calculates the pK_a values with 3.3 (0.4) and 8.9 (0.5), closely matching the experimental values of 3.1 and 8.99 [**?**]. Protonation states and pK_a values are shown in Figure S.I.19.

pkasolver-light identified the same states and calculates the pK_a values with 6.65 (0.5) and 8.6 (0.6).

Pyridine

A single protonation state was identified and pkasolver-epic calculates the pK_a value with 5.2 (0.2), closely matching the experimental pK_a value of 5.25 [**?**]. Protonation state and pK_a value is shown in Figure S.I.20.

pkasolver-light identified the same state and calculates the pK_a value with 5.0 (0.2).

Testing pkasolver-epic on the SAMPL6 molecules

The experimental and calculated pK_a values are shown in Table S.I.2. For 21 of the 24 molecules pkasolver-epic proposes too many protonation states in the investigate pK_a range [**?**]. This makes a direct comparison difficult. If the protonation state nearest to pH 7.4 is matched to the experimental pK_a values (shown in Table S.I.2 in red) the MAE of the predictions is 1.0 and the RMSE 1.4 pK_a units.

This set of molecules shows that the interplay between pkasolver and the sequential protonation state generation using Dimorphite-DL has to be improved. Currently, pkasolver tries to de- and protonate each protonation site proposed by Dimorphite-DL, irrespective of whether it is an acidic or basic site. Using a

[2] https://www.sigmaaldrich.com/deepweb/assets/sigmaaldrich/product/documents/315/570/d9628pis.pdf

heuristic to propose possible acidic and basic sites for a given pH range (something that `Dimorphite-DL` is able to do) would resolve some of this issue.

SAMPL6	Experimental pK_a			Calculated pK_a					
Molecule ID	pKa_1	pKa_2	pKa_3	pKa_1	pKa_2	pKa_3	pKa_4	pKa_5	pKa_6
SM01	9.53			6.8 (0.8)	8.9 (0.4)	10.3 (0.6)			
SM02	5.03			3.0 (0.4)	3.2 (0.5)	5.4 (0.7)	9.8 (0.35)		
SM03	7.02			3.5 (0.7)	4.7 (1.0)	7.5 (0.8)			
SM04	6.02			3.5 (0.7)	5.6 (1.0)	5.8 (0.4)	10.3 (0.3)		
SM05	4.59			4.4 (0.6)	5.7 (0.9)	10.1 (0.5)			
SM06	11.74	3.03		2.8 (0.2)	3.4 (0.3)	4.4 (0.7)	9.0 (0.5)		
SM07	6.08			3.5 (0.7)	5.7 (1.0)	5.9 (0.4)	10.5 (0.3)		
SM08	4.22			5.0 (0.7)	5.4 (1.1)	10.4 (0.5)			
SM09	5.37			3.3 (0.6)	3.6 (0.5)	6.1 (0.6)	10.2 (0.3)		
SM10	9.02			4.2 (0.9)	4.6 (0.9)	6.5 (0.5)	8.7 (0.6)	9.0 (0.4)	
SM11	3.89			3.2 (0.7)	3.6 (0.5)	4.3 (0.4)	8.9 (0.4)		
SM12	5.28			3.0 (0.5)	3.4 (0.5)	5.7 (0.7)	9.8 (0.3)		
SM13	5.77			3.7 (0.8)	4.1 (0.6)	6.3 (0.6)	10.2 (0.3)		
SM14	5.3	2.58		4.1 (0.7)	6.2 (0.4)	9.7 (0.4)			
SM15	8.94	4.7		5.7 (0.3)	8.9 (0.5)				
SM16	5.37	10.65		3.7 (0.7)	5.5 (0.4)	8.8 (0.6)			
SM17	3.16			3.1 (0.4)	3.7 (0.3)				
SM18	2.15	9.58	11.02	4.7 (0.6)	5.3 (1.2)	6.1 (0.7)	7.9 (0.6)	9.2 (0.5)	
SM19	9.56			4.2 (1.0)	6.5 (0.6)	9.3 (0.3)			
SM20	5.7			6.8 (0.4)					
SM21	4.1			3.3 (0.6)	4.8 (0.7)	8.3 (0.3)	9.4 (0.4)		
SM22	2.4	7.4		3.2 (0.4)	5.5 (0.4)				
SM23	5.5			2.9 (0.3)	3.4 (0.8)	4.1 (0.7)	5.7 (0.6)	9.0 (0.3)	9.7 (0.4)
SM24	2.6			3.5 (0.5)	5.4 (1.0)	8.9 (0.3)	9.1 (0.4)		

Table S.I.2. Experimental and calculated pK_a values for the 24 compounds of the SAMPL6 pK_a challenge [**?**]. pK_a values were calculated using `pkasolver-epic`. pK_a values and standard distribution (shown in parenthesis) are rounded to one significant digit. The pK_a value used to match the experimental pK_a value is shown in red.

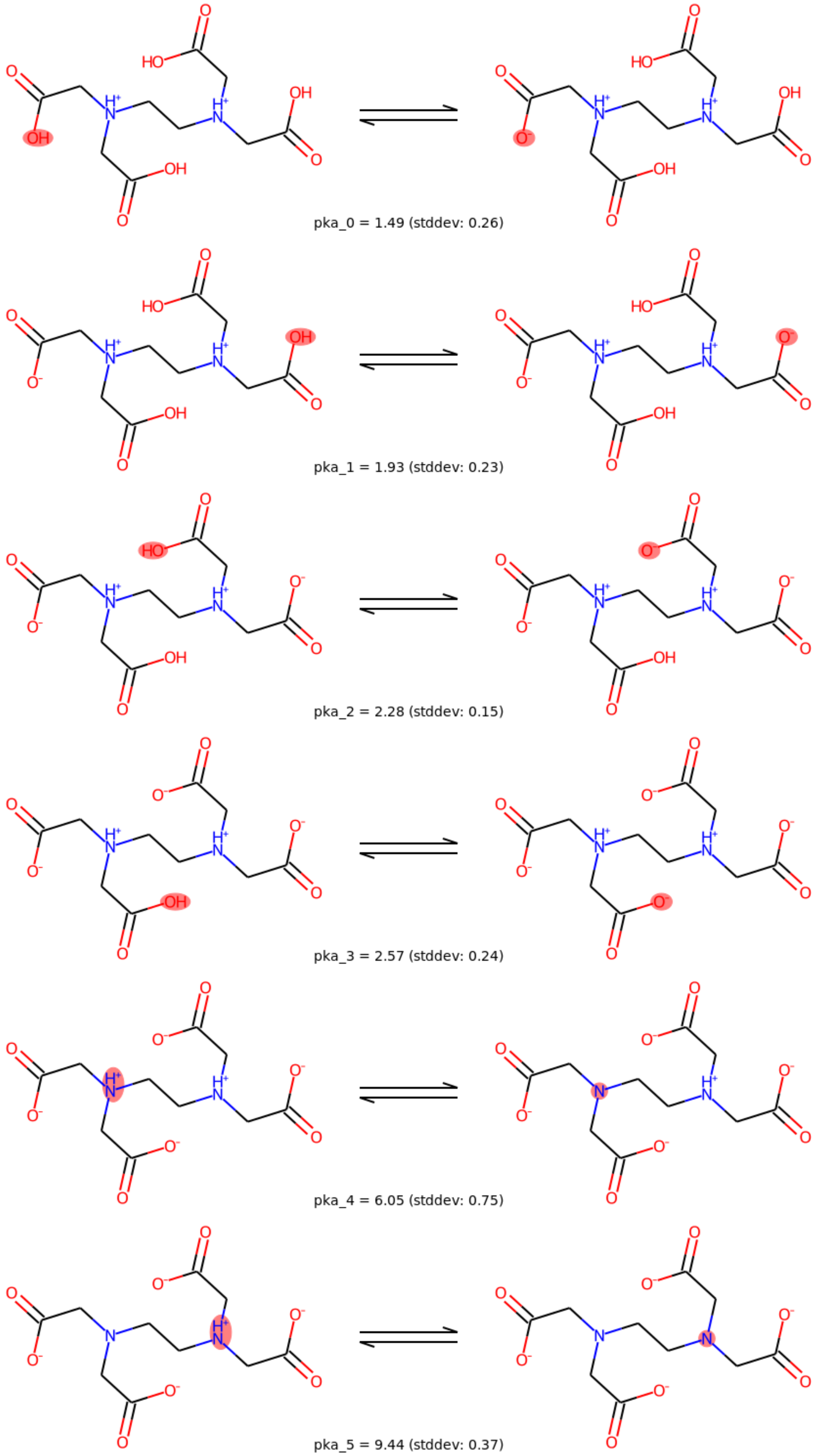

Figure S.I.9. Results are shown for a sequential pK_a prediction using `pkasolver-epic` for ethylenediaminetetraacetic acid (EDTA). For each protonation state the base-acid pair is shown and the consensus prediction for the pK_a value with the standard deviation is shown. The protonation site is highlighted for each protonation state.

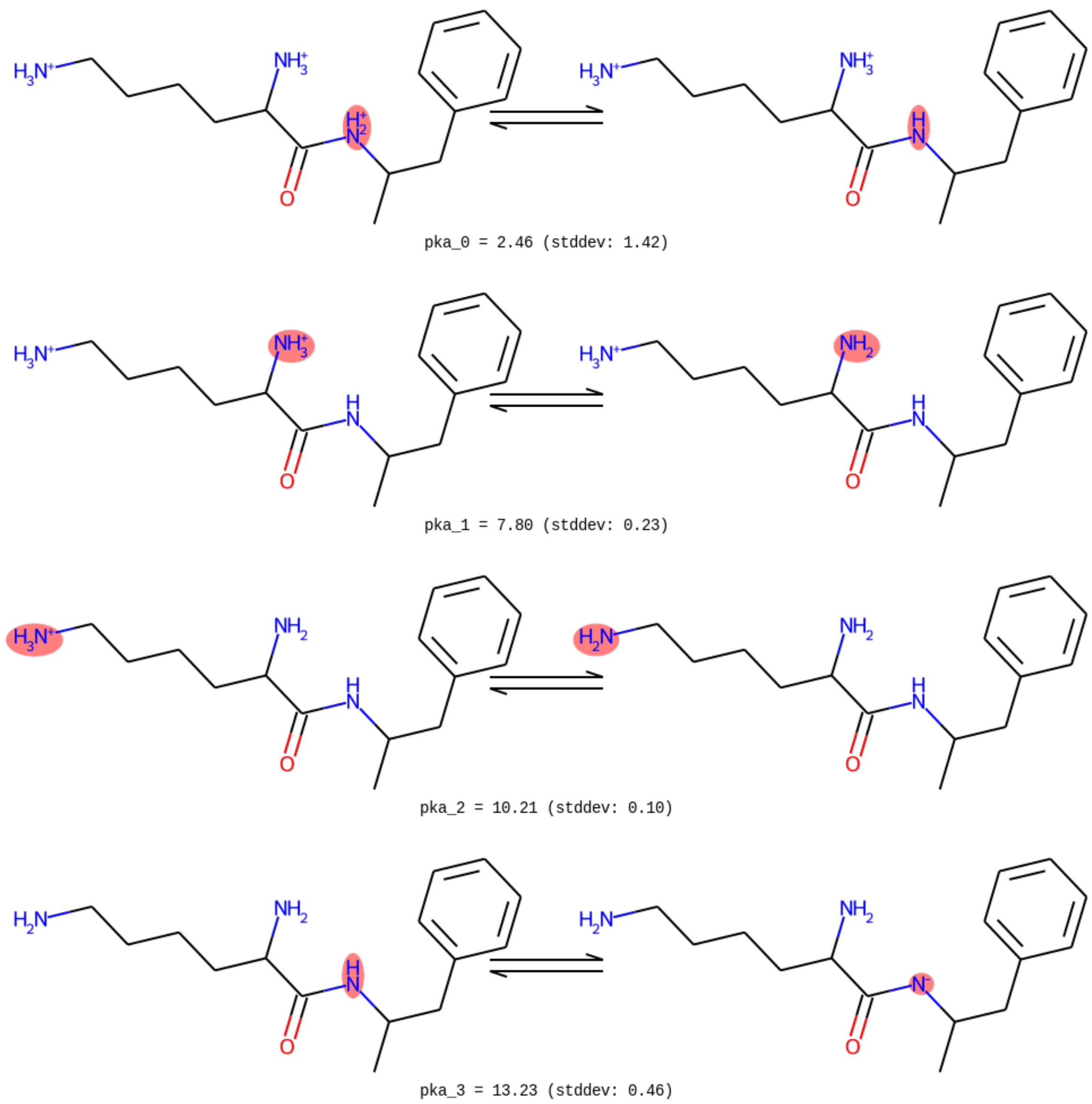

Figure S.I.10. Results are shown for a sequential pK_a prediction using `pkasolver-epic` for lisdexamfetamine.

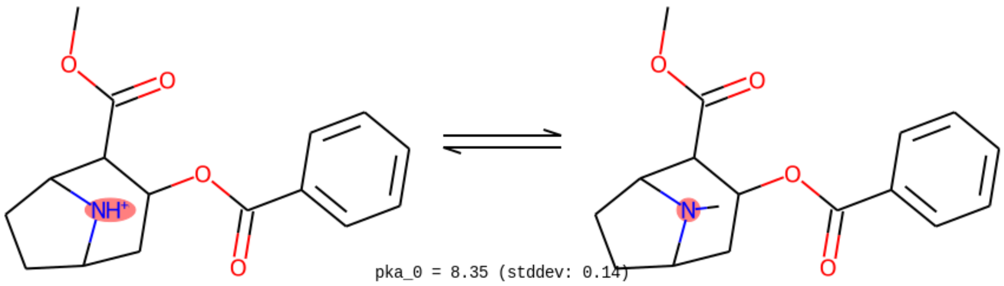

Figure S.I.11. Results are shown for a sequential pK_a prediction using `pkasolver-epic` for cocaine.

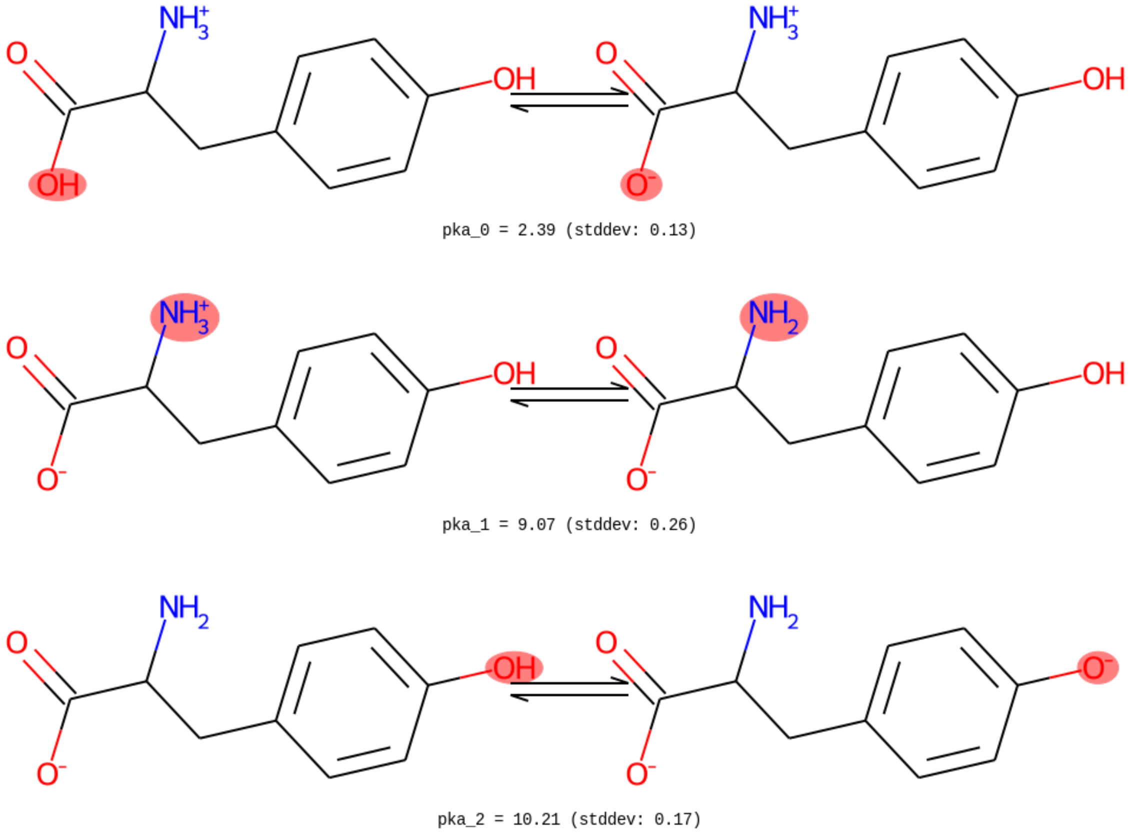

Figure S.I.12. Results are shown for a sequential pK_a prediction using `pkasolver-epic` for tyrosine.

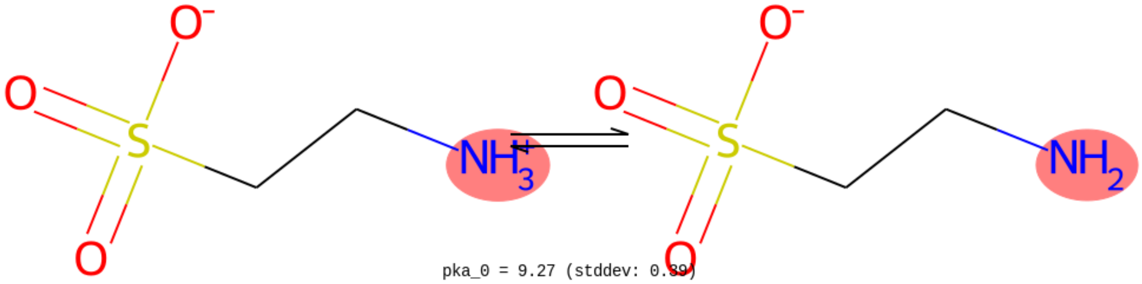

Figure S.I.13. Results are shown for a sequential pK_a prediction using `pkasolver-epic` for taurine.

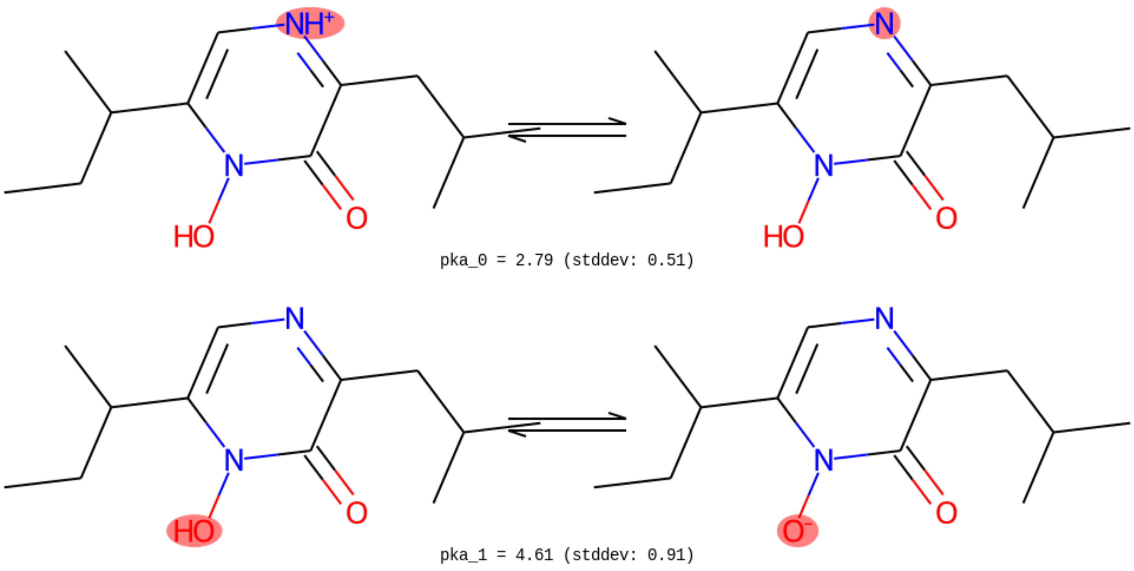

Figure S.I.14. Results are shown for a sequential pK_a prediction using `pkasolver-epic` for aspergillic acid.

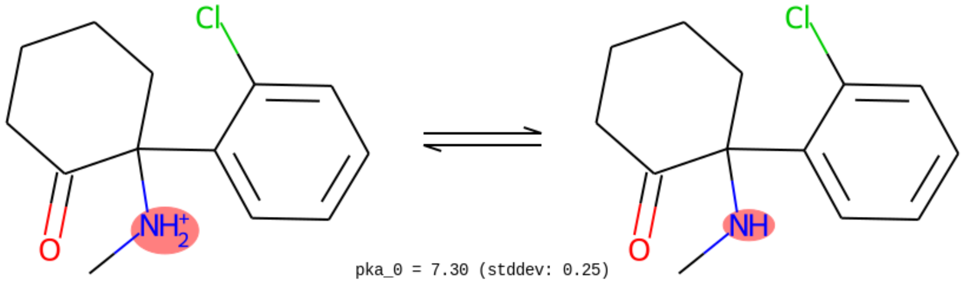

Figure S.I.15. Results are shown for a sequential pK_a prediction using `pkasolver-epic` for ketamine.

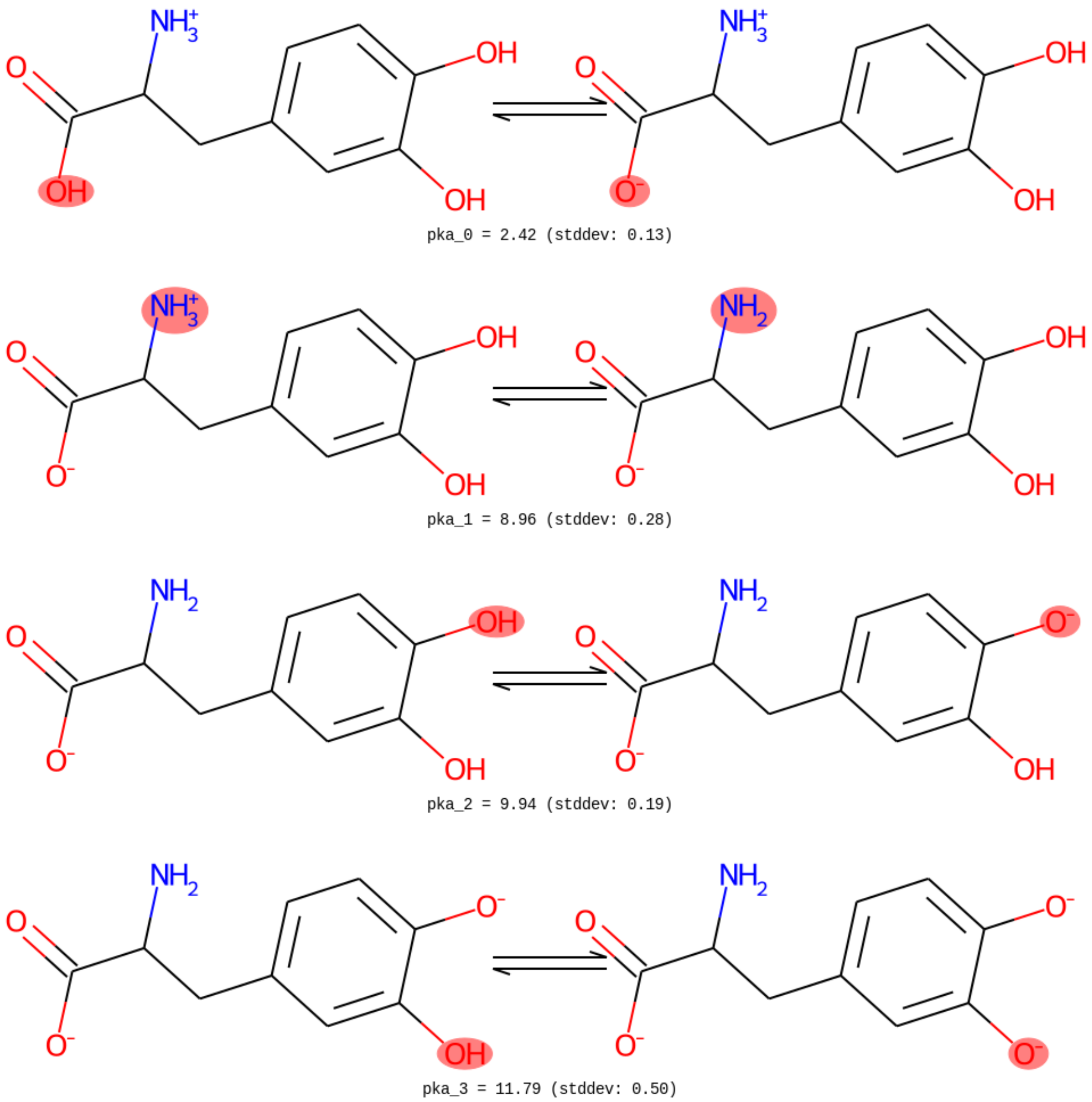

Figure S.I.16. Results are shown for a sequential pK_a prediction using `pkasolver-epic` for levodopa.

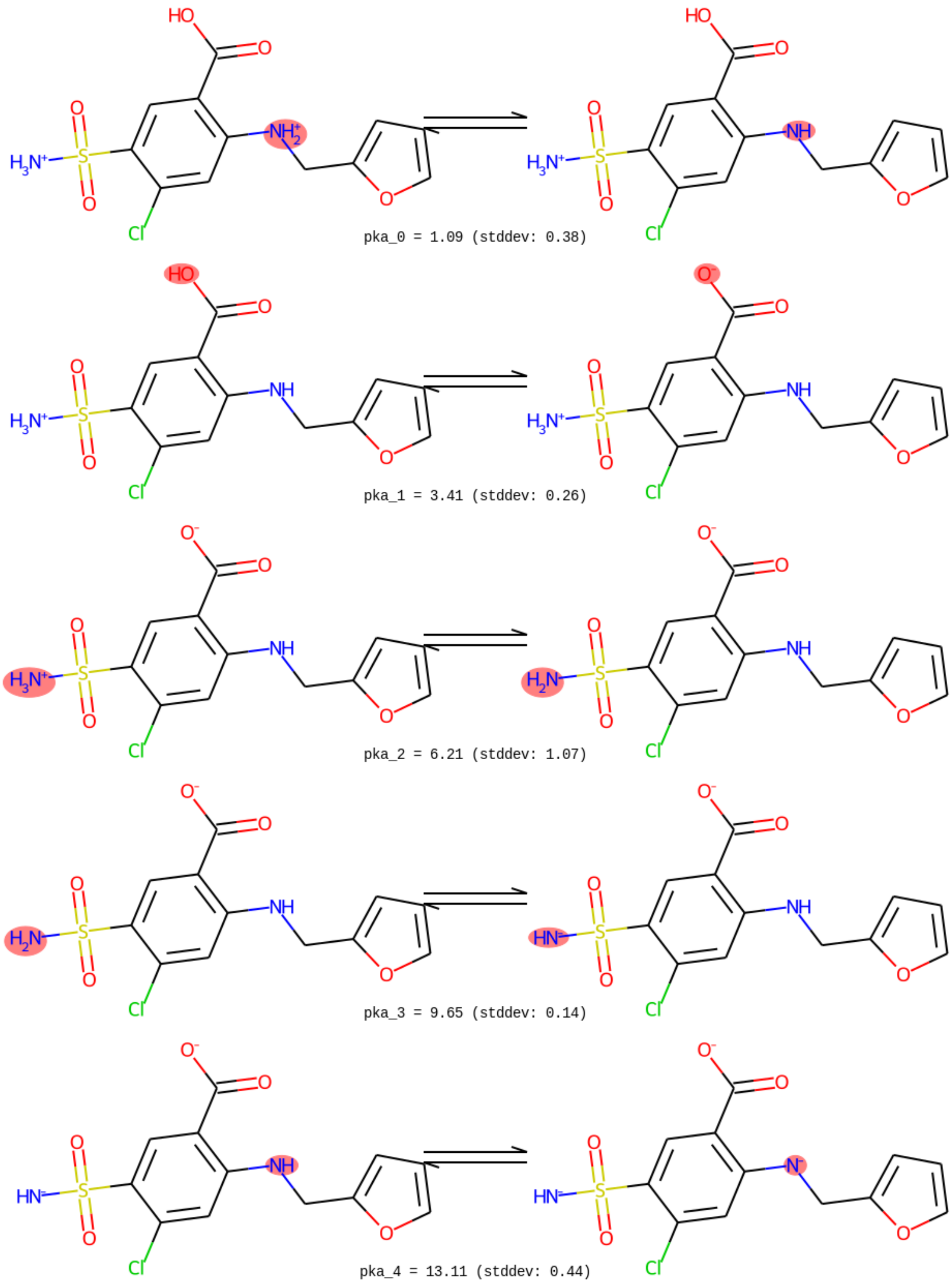

Figure S.I.17. Results are shown for a sequential pK_a prediction using `pkasolver-epic` for furosemide.

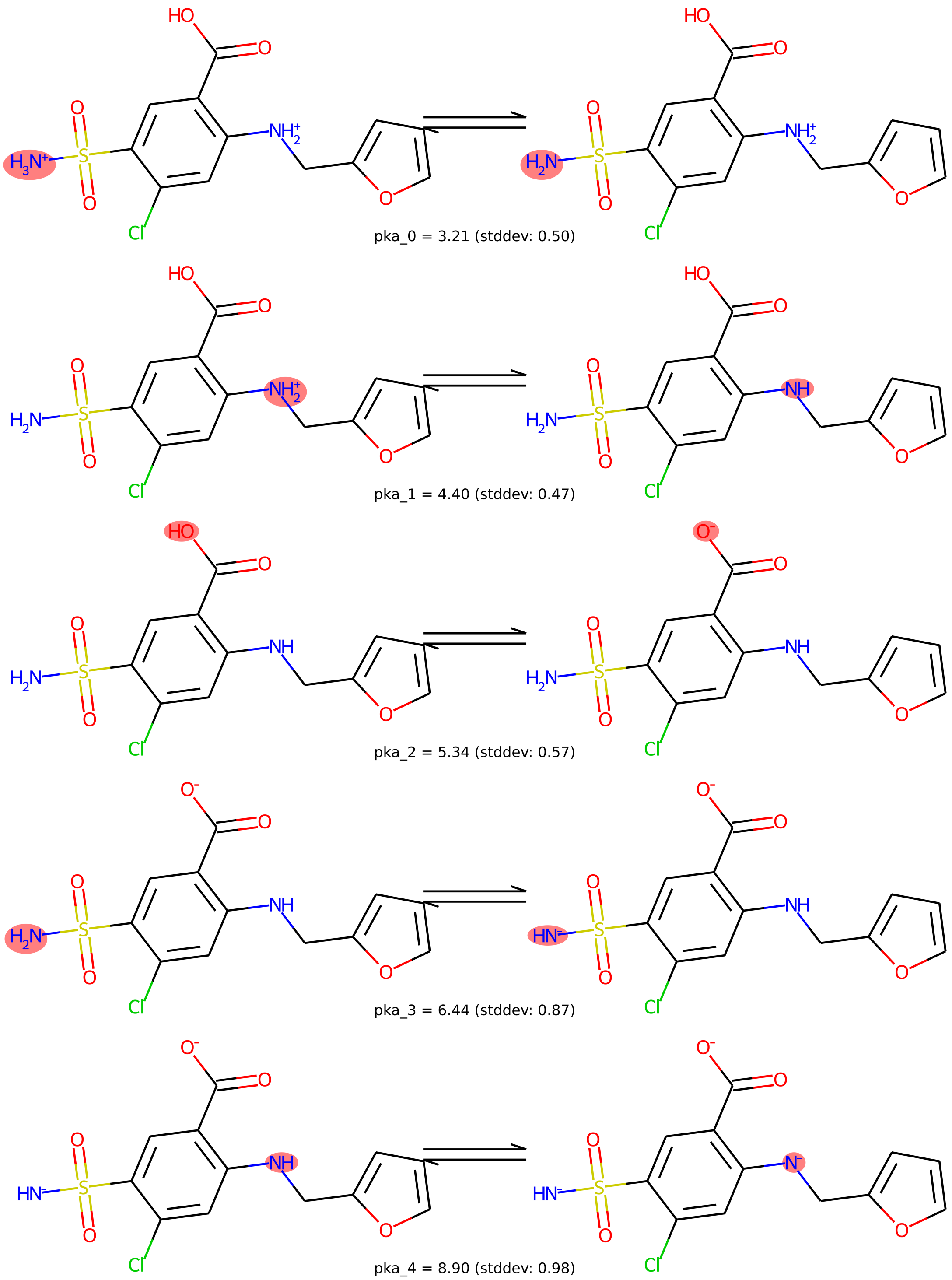

Figure S.I.18. Results are shown for a sequential pK_a prediction using `pkasolver-light` for furosemide.

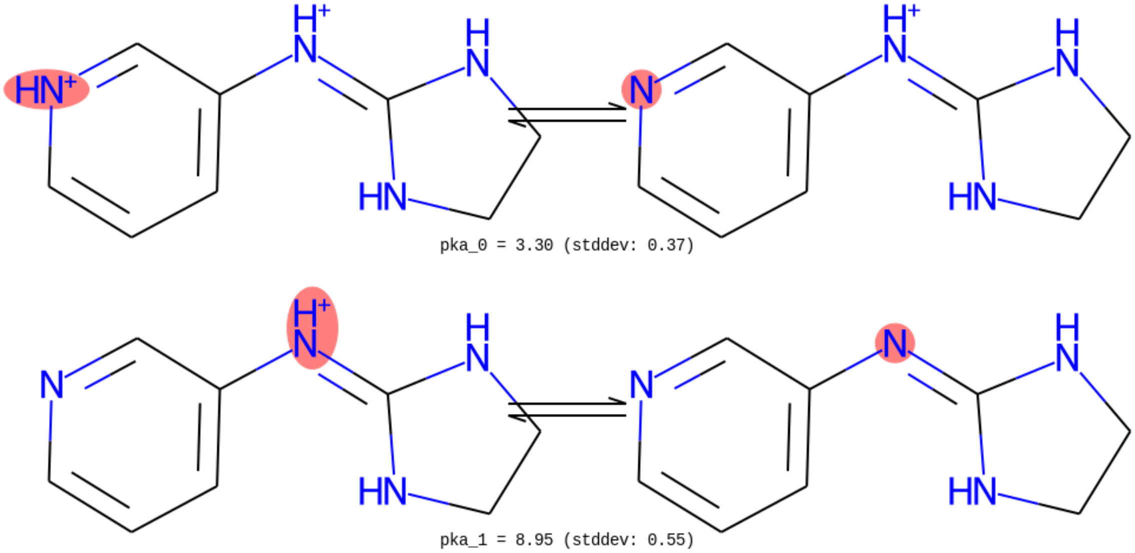

Figure S.I.19. Results are shown for a sequential pK_a prediction using `pkasolver-epic` for an aryl guanidine (SMILES: C1CNC(N1)=NC1=CC=CN=C1).

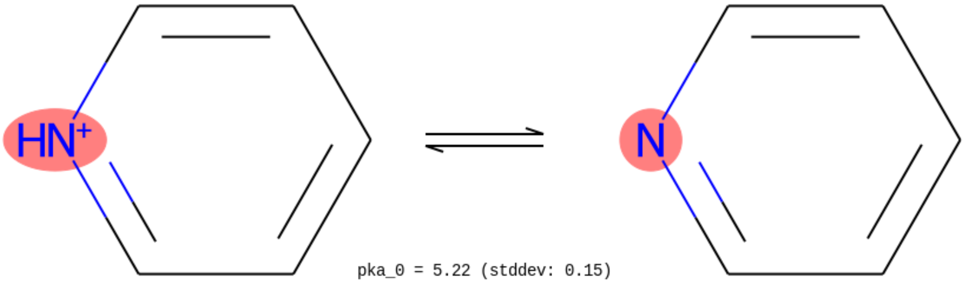

Figure S.I.20. Results are shown for a sequential pK_a prediction using `pkasolver-epic` for pyridine.
